# Postneonatal Mortality and Liver Changes in Cloned Pigs Associated with Human Tumor Necrosis Factor Receptor I-Fc and Human Heme Oxygenase-1 Overexpression

**DOI:** 10.1155/2017/5276576

**Published:** 2017-04-19

**Authors:** Geon A. Kim, Jun-Xue Jin, Sanghoon Lee, Anukul Taweechaipaisankul, Hyun Ju Oh, Joing-Ik Hwang, Curie Ahn, Islam M. Saadeldin, Byeong Chun Lee

**Affiliations:** ^1^Department of Theriogenology and Biotechnology, Research Institute for Veterinary Science, College of Veterinary Medicine, Seoul National University, Gwanak-ro 151-742, Republic of Korea; ^2^Graduate School of Medicine, Korea University, 73 Inchon-ro, Seongbuk-gu, Seoul 136-705, Republic of Korea; ^3^Designed Animal Resource Center and Biotransplant Research Institute, Seoul National University Green BioResearch Complex, Gangwon-do, Republic of Korea; ^4^Division of Nephrology, Seoul National University, College of Medicine, Seoul, Republic of Korea; ^5^Transplantation Center, Seoul National University Hospital, Seoul, Republic of Korea; ^6^Department of Animal Production, College of Food and Agricultural Sciences, King Saud University, Riyadh 11451, Saudi Arabia; ^7^Department of Physiology, Faculty of Veterinary Medicine, Zagazig University, Zagazig 44519, Egypt

## Abstract

Soluble human tumor necrosis factor (shTNFRI-Fc) and human heme oxygenase 1 (hHO-1) are key regulators for protection against oxidative and inflammatory injury for xenotransplantation. Somatic cells with more than 10 copy numbers of shTNFRI-Fc and hHO-1 were employed in somatic cell nuclear transfer to generate cloned pigs, thereby resulting in seven cloned piglets. However, produced piglets were all dead within 24 hours after birth. Obviously, postnatal death with liver apoptosis was reported in the higher copy number of shTNFRI-Fc and hHO-1 piglets. In liver, the transcript levels of ferritin heavy chain, light chain, transferrin, and inducible nitric oxide synthase were significantly highly expressed compared to those of lower copy number of shTNFRI-Fc and hHO-1 piglets (*P* < 0.05). Also, H_2_O_2_ contents were increased, and superoxide dismutase was significantly lower in the higher copy number of shTNFRI-Fc and hHO-1 piglets (*P* < 0.05). These results indicate that TNFRI-Fc and hHO-1 overexpression may apparently induce free iron in the liver and exert oxidative stress by enhancing reactive oxygen species production and block normal postneonatal liver metabolism.

## 1. Introduction

Implication of somatic cell nuclear transfer (SCNT) for the production of genetically modified pigs is widely accepted [1]. Especially, as organ donors for xenotransplantation, transgenic pigs have been successfully produced due to the similarities in anatomical organ size, physiological features, and immunological features with human [2]. Since the first report for Gal deficiency pig production using SCNT [3], various transgenic pigs with multiple genes modifications including CD39, CD55, CD59, endothelial protein C receptor, and thrombomodulin were generated for xenotransplantation [4]. With this impressive success, the phenotype and characteristics of transgenic pigs for the practical use should be investigated. Generally, transgene copy number can influence transgene expression in mouse [5] as well as in pigs [6]. Furthermore, previous studies on transgenesis indicated that copy number of transgene could affect the clinical phenotype and mortality [7, 8].

Tumor necrosis factor (TNF) is an inflammatory cytokine produced by macrophages or monocytes during acute inflammation and is responsible for a diverse range of signaling events within cells [9]. In xenograft rejection, human TNF-*α* can induce acute inflammatory reaction in porcine cells [10, 11]. Transgenic mice expressing soluble human TNF receptor type I protein with human IgG Fc (shTNFRI-Fc) were produced and were used for studying pathogenesis of bone loss induced by estrogen deficiency [12]. Meanwhile, previously transgenic pig, in which shTNFRI-Fc is driven by cytomegalovirus promoter, achieved shTNFRI-Fc expression and suppressed porcine endothelial cell activations [13]. It has been also demonstrated that TNF and its receptor has been implicated as an important pathogenic player in patients with liver diseases [14], although it is required for normal hepatocyte proliferation during liver generation [15, 16].

Heme oxygenase-1 (HO-1) is the enzyme that degrades heme into biliverdin, carbon monoxide (CO), and free iron. Because all three end products have antioxidative, anti-inflammatory, and antiapoptotic effects [17], it has been demonstrated that human HO-1 transgenic pigs showed resistance against xenograft rejection [18] and protect xenografts when exposed to oxidative stress [19]. Besides the protective role of HO-1 against heme mediated cell injury, excess heme-iron has been implicated in liver damage [20]. Therefore, HO-1 is a double-edged sword as well as shTNFRI.

For xenotransplantation, blocking TNF-*α* by the overexpression of shTNFRI-Fc and combinatorial expression of HO-1 could reduce acute graft rejection and immediate blood mediated immune response for preventing inflammatory reaction, cell activation, and apoptosis [21]. However, the characteristics and clinical feature of transgenic pig with excess shTNFRI-Fc and HO-1 have not been reported. Our lab has used a unique cell line of shTNFRI-Fc and HO-1 pig for SCNT that have only 15 copies of the shTNFRI-Fc and HO-1 genes. In the present study, we focus on the function of shTNFRI-Fc and HO-1 in transgenic pigs to study the mechanism of shTNFRI-Fc and HO-1 on liver metabolism. Therefore, the aim of the present study is to characterize pathological phenotype of shTNFRI-Fc and HO-1 transgenic pig models, which exhibited early postnatal mortality and liver damage.

## 2. Materials and Methods

All experiments were performed following Seoul National University' Institutional Animal Care and Use Committee approved protocol (SNU-141120-8) in accordance with the Guide for the Care and Use of Laboratory Animals. Procedures for animal treatment and surgery have been previously published.

### 2.1. Cell Preparation and Somatic Cell Nuclear Transfer

From previously generated C15-shTNFRI-Fc/hHAHO-1 transgenic pigs, neonatal fibroblasts were isolated and cultured in DMEM media (Invitrogen, Carlsbad, CA, USA) [21]. SCNT was performed as recently described using in vitro matured oocytes [22]. From porcine ovaries, cumulus oocyte complexes with homogenous cytoplasm and three or more layers of cumulus cells were recovered and cultured for 44 h for oocyte in vitro maturation. Briefly, bisbenzimide stained nucleus and polar body were aspirated in Tyrode's albumin lactate pyruvate medium droplets and rechecked under ultraviolet radiation to confirm complete nuclear materials removal. A fibroblast was placed in the perivitelline space in close contact with the oocyte membrane to form a couplet. After cell injection, fusion was induced with a single DC pulse of 200 v/mm for 30 *µ*s using an electrical pulsing machine (LF101; Nepa Gene, Chiba, Japan). Reconstructed couplets were activated with a single DC pulse of 1.5 kV/cm for 60 *µ*s using a BTX ElectroCell Manipulator 2001 (BTX Inc., San Diego, CA). Then, reconstructed embryos were cultured in porcine zygote medium-5 at 38.5°C in a humidified atmosphere with 5% O_2_, 5% CO_2_, and 90% N_2_. In total, 368 reconstructed embryos were surgically transferred into both oviduct of six naturally synchronized sows. All recipients were assessed for pregnancy diagnosis using ultrasound scanning between days 25 and 35 of gestation. After 114 days of embryo transfer (ET), seven piglets were delivered by the Cesarean section.

### 2.2. Genotyping of Transgenic Clone Pigs

Genomic DNA isolated from the TG pig tail was used for identification of target gene expression by PCR. The primers for the transgenes shTNFRI-Fc and hHAHO-1 were identified in the previous study [21]. All primers were obtained from Bioneer Corp. (Bioneer Corp., Daejeon, South Korea) and PCR products of both shTNFRI-Fc and hHAHO-1 were visualized under UV light after electrophoresis on 1% agarose gel stained with ethidium bromide and demonstrated single products of the predicted sizes.

### 2.3. Cell Viability

To investigate the function of the transgenes shTNFRI-Fc and hHAHO-1 in cloned piglets, samples were isolated and tail-skin derived fibroblasts were plated and were seeded in 24-well plates (1 × 10^5^ cells/well) and cultured with fresh DMEM medium containing 10% FBS. For oxidative stimulation, H_2_O_2_ of 400 *µ*M concentration were applied for 1 hr. For apoptotic stimulation, tumor necrosis factor-*α* (TNF-*α*; eBioscience, CA, USA) and cycloheximide (CHX; Sigma, MO, USA) was used. The concentrations of hTNF-*α* 20 ng/ml and CHX 10 *µ*g/ml were determined based on a previous study [19]. Cell viability was detected by Cell Counting Kit-8 (CCK-8; Dojindo Laboratories, Kumamoto, Japan) assay according to the manufacturer's instructions. After oxidative and apoptotic stimulation, CCK-8 solution of one-tenth of the volume of the medium was added to each well of the cell plate. The fibroblasts were incubated for 1 hr after the addition of the CCK-8 solution. The absorbance was measured at 450 nm using a microplate reader.

### 2.4. Copy Number Calculation

Transgene copy number integrated into the genome was calculated by semiquantitative polymerase chain reaction (PCR). In brief, Genomic DNA was isolated from cells or tissues of transgenic pigs by DNA lysis buffer (10 mM Tris pH 8.5, 5 mM EDTA, 0.2% SDS, 200 mM NaCl, and 100 *µ*g/ml proteinase K). 0.1 *µ*g genomic DNA was applied for PCR with primers (forward 5′-AGGTGGAGATCTCTTCTTGCAC-3′ and reverse 5′-TACGTGCTGTTGTACTGCTCCT-3′). PCR product size was 529 bp for TNFRI cDNA. By designating 2.7 × 10^9^ bp as the haploid content of the pig genome, a single copy standard can be calculated as 0.0098 pg of transgene DNA added to 0.1 *µ*g genomic DNA. To obtain results from standard PCR, pcDNA3.1/sTNFR1-Fc-F2A-HO1 was used for template. PCR conditions were conducted for 25 cycles at the condition of denaturation at 94°C for 30 sec, annealing at 57°C for 30 sec, and extension at 72°C for 40 sec. 10 *µ*l of PCR products was loaded onto a 1.5% agarose gene. Generation of a 529-bp nucleotides fragment was confirmed by ethidium bromide staining.

### 2.5. Relative Quantitative mRNA Transcript Analysis by Real-Time PCR in Liver

Analysis of mRNA transcripts of HO-1, transferrin, inducible NOS, ferritin heavy chain, and ferritin light chain was performed using qPCR. The oligonucleotide primer sequences used are listed in Table 1. Total RNA was extracted by using RNA extraction kit according to the manufacturer's instructions. After quantification of RNA concentration using a NanoDrop 2000 Spectrophotometer (Thermo Fisher Scientific, Wilmington, DE, USA), complementary DNA (cDNA) was produced using cDNA synthesis Platinum Master Mix (GenDEPOT, Barker, TX, USA). GAPDH was used as internal control to normalize the relative differences in the amount of cDNA in each sample. Using a SYBR Premix Ex Taq (TaKaRa, Otsu, Japan), real-time PCR was conducted with the ABI Prism 7300 system (Applied Biosystems, Waltham, MA, USA) using a PCR plate (Micro-Amp Optical 96-Well Reaction Plate, Singapore). Thermocycler conditions included an initial denaturation for 10 min at 95°C followed by 40 cycles consisting of denaturation for 15 s at 95°C, annealing for 1 min at 60°C, and extension for 1 min at 72°C. Data were collected and quantitatively analyzed on an ABI PRISM 7300 sequence detection system (Applied Biosystems). All values were expressed as fold increase or decrease relative to the expression of GAPDH.

Each transcript was relatively quantified in three replicates by calculation using 2^−ΔΔCt^ method for comparison of relative mRNA quantification in each sample after normalizing to the housekeeping gene GAPDH.

### 2.6. TNFRI, H_2_O_2_, and Superoxide Dismutase Analysis Using ELISA

For analysis of TNFRI, H_2_O_2_, and superoxide dismutase protein in liver of dead cloned piglets, liver lysate was weighed followed by homogenization in 200 *µ*l buffer (0.05 M potassium phosphate and 0.1 Mm EDTA, pH 7.8) and centrifuged at 15,000 ×g for 30 min at 4°C. Enzyme-linked immunosorbent assay (ELISA) was carried out using specific ELISA kits for shTNFRI- (R&D system, MN, USA), H_2_O_2_, and superoxide dismutase (Koma-biotech, Seoul, Korea) according to the manufacturer's recommendation.

### 2.7. Western Blot Analysis

Tissues were finely grinded by homogenizer and washed with phosphate buffer solution (PBS, pH 7.4) and lysed with ice-cold lysis buffer (50 mM Tris-HCl, pH 7.2; 150 mM NaCl; 1 mM EDTA; 1% Triton X-100; 0.1% aprotinin; 0.1% SDS; 1 mM PMSF). The lysate was clarified by centrifugation (13,000 rpm, 10 min at 4°C). 10 *µ*g of the protein from the supernatant was fractionated by 10% SDS-PAGE and electrotransferred onto a PVDF membrane. The blots were blocked in 5% skim milk at 4°C overnight. In the next day, the membrane was incubated with mouse anti-HO-1 monoclonal antibody (1 : 2500 dilution with 5% skim milk Tris-buffered saline containing 0.1% Tween 20 (TBST)) for 2 hr. Then membranes were washed three times each for 10 min with TBST incubated with horseradish peroxidase conjugated goat anti-mouse IgG (1 : 5000) for 1 hr. Afterward, the membranes were developed with enhanced chemiluminescence reagents and the positive signals were exposed to X-ray films. The densities of the immunoblots were scanned with image acquisition system (VilberLourmat, Fusion SL3500).

### 2.8. Histopathology Examination

After fixing the liver fragment in 10% neutral formalin, embedding in paraffin then was stained with standard hematoxylin and eosin (H&E) procedures. Bright field images were obtained with a Leica DMI 6000B microscope using a DFC350 camera.

### 2.9. Statistical Analysis

The experiments were repeated at least three times for each experiment. All statistical analyses were performed using Prism program. Cell viability, gene expression, shTNFRI, H_2_O_2_, and SOD ELISA were compared by one-way ANOVA with Dunn's Multiple Comparison Test.

## 3. Results

### 3.1. The Production of Transgenic Pigs

**Figure 1:**
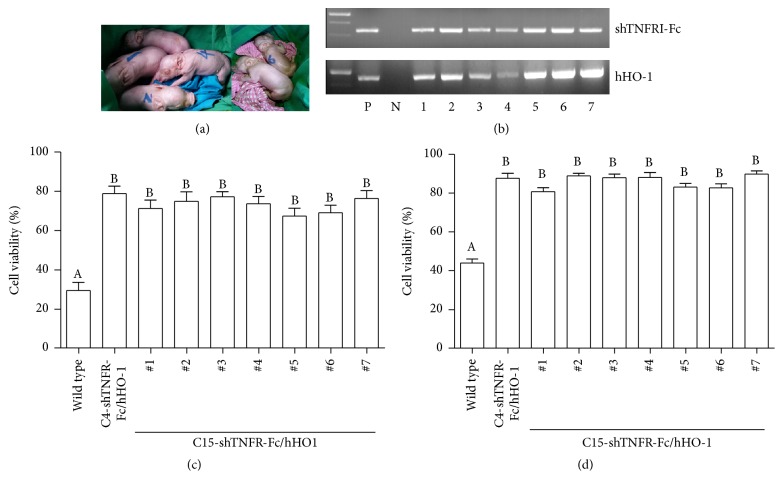
Demonstration of transgenesis in cloned pigs produced by SCNT. (a) Picture of cloned pigs after birth. Birth weight piglet #1: 1030 g, piglet #2: 750 g, piglet #3: 764 g, piglet #4: 640 g, piglet #5: 453 g, piglet #6: 406 g, and piglet #7: 400 g. (b) PCR analysis on genomic DNA isolated from the produced transgenic animals (#1~#7) and nontransgenic control, wild type. PCR with shTNFRI-Fc (upper panel) and HO-1 (lower panel) specific primers demonstrated the presence of the shTNFRI-Fc and hHO-1 transgene. P, plasmid control; N, negative control. (c) Resistance of fibroblasts from transgenic pigs against oxidative stress. The fibroblasts of 1 × 10^4^ cells per well from transgenic pig were stimulated with hTNF-*α* (20 ng/ml) and CHX (10 micrograms/ml) stimulation for 15 h and the viability was determined by CCK-8. *P* values versus wild type pig fibroblasts (^*∗*^*P* < 0.05). The data shown were obtained in six replicates. (d) Cell viability of fibroblasts against apoptotic stimulation. Fibroblasts of 1 × 10^4^ cells per well from each TG pig were treated with H_2_O_2_ (400 *μ*M) for 1 h. *P* values versus wild type pig fibroblasts (^*∗*^*P* < 0.05). The data shown were obtained in six replicates. Columns carrying different letters are statistically different at *P* < 0.05.

Fibroblasts from white Yucatan pig (O-type blood) with ubiquitous expression of the shTNFRI-Fc and hHAHO-1 were used as donor cells for SCNT. On average, 368 reconstructed embryos were transferred into each of the 6 naturally synchronized recipient pigs. Four of them became pregnant by ultrasound scanning, and only one farrowed 7 live male piglets at term (Table 2, Figure 1(a)). Other three recipients became aborted. At birth, one could not breathe and showed abnormality including hind limb luxation (piglet #7, data not shown). Although six piglets could breathe well, they could not eat and stand themselves. One day later, all piglets showed clinical abnormal symptoms dyspnea. Although the oxygen was supplied, all produced piglets died. The insertion of shTNFRI-Fc and hHAHO was confirmed in the genome of produced piglets (Figure 1(b)).

### 3.2. Cell Viability against Oxidative Stress and Apoptotic Stimulation

Fibroblasts were isolated from the produced 7 piglets and cell viability after hTNF-*α* (20 ng/ml) and CHX (10 micrograms/ml) stimulation or H_2_O_2_ (400 *µ*M) were determined. The cell viability from produced 7 piglets (C15-shTNFRI-Fc/hHO-1) was significantly higher than those of wild type in both apoptotic and oxidative stress (Figures 1(c) and 1(d); *P* < 0.05).

### 3.3. Copy Number of Gene in Transgenic Pigs

**Figure 2:**
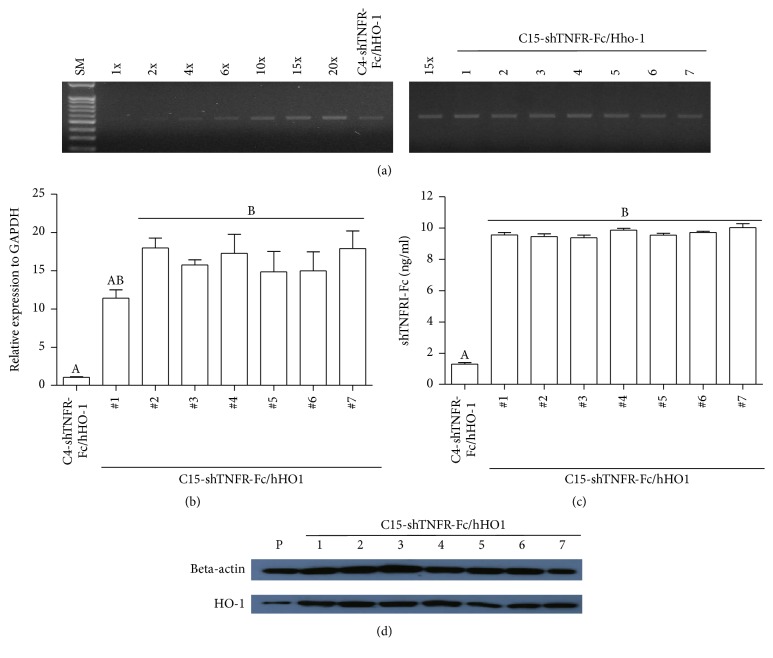
Copy number, hHO-1 and shTNFRI-Fc expression level of transgenic pigs. (a) While a C4-shTNFRI-Fc/hHO-1 piglet have at least 4 copies in genome, all C15-shTNFRI-Fc/hHO-1 piglets harbor 15 copies of the hHO1 and shTNFRI-Fc transgene in their genome. Genomic DNA from transgenic pig was extracted and analyzed using gradient PCR. (b) Relative expression of HO-1 mRNA in liver was detected by real-time PCR. The experiment is performed in 9 replications and data are presented as mean values ± standard deviation. Different superscript means significance (*P* < 0.05). (c) Quantitative analysis of shTNFRI-Fc analysis in liver was performed using ELISA. The experiment is performed in eight replications and data are presented as mean values ± standard deviation. (d) HO-1 in the liver protein lysate of transgenic pigs. Relative expression of HO-1 protein in liver of transgenic pigs was detected by Western blot. Anti-*β*-actin staining was used as a loading control. A clear overexpression of shTNFRI-Fc/hHAHO1/B KO compared to shTNFRI-Fc/hHAHO1/A KO is evident in all transgenic pigs.

To determine copy number of transgenes in cloned pigs, genomic PCR was performed. While a C4-shTNFRI-Fc/hHO-1 piglet without clinical abnormal symptoms has at least 4 copies in genome, all C15-shTNFRI-Fc/hHO-1 transgenic piglets harbor 15 copies of the hHO-1 and shTNFRI-Fc transgene in their genome (Figure 2(a)).

### 3.4. Expression of shTNFRI-Fc and hHAHO in Liver

The relative quantification of hHO-1 was significantly increased in the liver of seven C15-shTNFRI-Fc/hHO-1 pigs compared to that of a C4-shTNFRI-Fc/hHO-1 pig. The concentration of shTNFRI-Fc in transgenic pig derived liver was significantly higher than those of normal pig without clinical symptoms (Figure 2(c); *P* < 0.05). The protein hHO-1 concentrations of liver extract of seven C15-shTNFRI-Fc/hHO-1 pigs were higher than that of a C4-shTNFRI-Fc/hHO-1 pig (Figure 2(d); *P* < 0.05).

### 3.5. Histological Examination of the Liver in Transgenic Pigs

Autopsy was performed immediately after death and the liver of C15-shTNFRI-Fc/hHO-1 pigs was examined. Gross appearance of the liver showed severe congestion and microscopic examination of affected liver slides revealed severe multifocal hemorrhagic necrosis (Figures 2(a) and 2(b)).

### 3.6. Gene Expression, H_2_O_2_, and Superoxide Dismutase in the Liver of Transgenic Pigs

**Figure 3:**
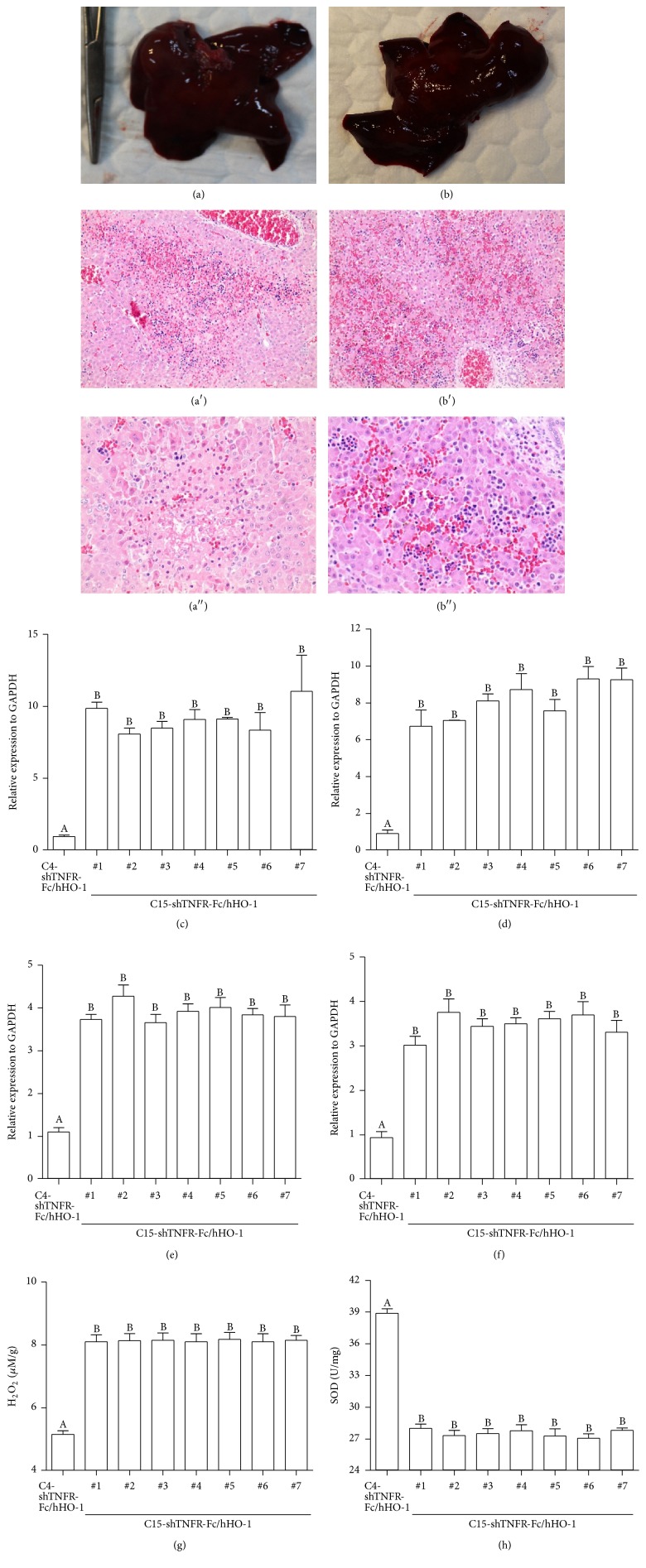
Transgenic pig harboring 15 transgenes shows accumulation of hemorrhagic apoptosis in liver. After death of transgenic pigs, autopsy was performed. (a)-(b) Representative gross images of liver of C15-shTNFRI-Fc/hHO-1 pigs. (a′)-(b′) Representative images of hematoxylin and eosin (H&E) stating of liver. Original magnification, 200x. (a′′)-(b′′) Original magnification, 400x. (c)–(f) The expression of several genes in liver of a C4-shTNFRI-Fc/hHO-1 transgenic pig and seven C15-shTNFRI-Fc/hHO-1 pigs. (c) Ferritin heavy chain, (d) ferritin light chain, (e) transferrin, and (f) inducible NOS. Data were shown as means ± standard deviations of six replicates. Data followed by different letters are significantly different (*P* < 0.05). (g)-(h) The quantitative analysis of H_2_O_2_ (g) and superoxide dismutase (h) in produced piglets liver. Data were shown as means ± standard deviations of six replicates. Data followed by different letters are significantly different (*P* < 0.05).

The relative ferritin heavy chain, light chain, transferrin, and inducible nitric oxide synthase mRNAs levels in 15 copy number transgenic cloned pigs are significantly increased compared that of lower copy number of transgenic pig (Figures 3(c)–3(f); *P* < 0.05). While the liver of transgenic pigs harboring 15 copy number exhibited the higher H_2_O_2_, SOD contents of transgenic pigs harboring 15 copy number were significantly lower (Figures 3(g) and 3(h); *P* < 0.05).

## 4. Discussion

Using SCNT, transgenic cloned pigs were successfully produced without gross abnormality. However, they could not eat well; during the early postnatal period, overall similar clinical symptoms including dyspnea were evident. In view of the protective role of HO-1 and TNFRI-Fc in transgenic pigs, we had expected that the excess of these factors in the proposed transgenic pigs could be maintained and used for xenotransplantation.

The founder shTNFRI-Fc transgenic pig and its offspring and HO-1 transgenic pig and its offspring are all healthy, without any sign of side effect from systemic expression of the transgenic protein. However, it has been reported that hepatocyte dysfunction could occur as a result of accumulation of free heme and high levels of serum TNF-*α* during malaria [23, 24]. It could be assumed that excess TNF-*α* expression stimulates the proinflammatory response, thereby causing hepatocyte death.

Moreover, increased HO-1 levels also may cause liver damage in pigs [25]. HO-1 has been thought to be solely involved in the catabolism of heme from red blood cells or denatured heme proteins. The main biological function of HO-1 is to avoid the deleterious accumulation of free heme. However, the overexpression of HO-1 in pig is not investigated yet. In the present study, we provide the experimental evidence that excess copy number of HO-1 can induce HO-1 overexpression and could affect the liver apoptosis. Until recently, HO-1 gene dosage appears to be linked to embryonic survival. The mother of HO-1 deficient patient especially had experienced several fetal deaths [26]. It has been evidenced that polymorphisms in the human HMOX1 gene are highly associated with an increased risk for recurrent miscarriage [27]. Notably, long-term overexpression of HO-1 can enhance iron loading and promote tau aggregation in brain [28].

HO-1 breaks down the porphyrin ring to yield equimolar amounts of biliverdin, free iron (Fe^2+^), and carbon monoxide (CO). All the degradation products during this process were considered as toxic [29]. Free iron is sequestered in ferritin or released with the aid of iron transporter. Among these products, free iron (II) can cause severe oxidative stress by the generation of reactive molecules, such as the highly reactive hydroxyl radical [30]. Thus iron is generally considered a powerful prooxidant and harmful to cells.

Metabolic syndrome has been ascribed to mechanisms that include a change in the levels of cellular heme-dependent proteins that increase oxidative stress [31]. Excess heme-iron has been implicated in liver damage [20]. Also, heme iron-mediated oxidative stress magnifies the adverse effect by introducing inflammation in liver [32]. Previous studies demonstrated that increase of HO-1 activity is accompanied by enhanced ferritin synthesis, whereas inhibition of HO activity could induce a decrease in ferritin levels [33–35]. It has been known that ferritin synthesis could be prevented by chelating iron, and it fails to protect against iron-mediated oxidative stress [36–38]. In the present study, it seems that excess HO expression accelerate the transferrin expression. Free iron could induce the expression of heavy chain ferritin [39, 40], which constitutes an ATPase dependent iron pump that decreases the level of intracellular Fe^2+^. Iron in heme is necessary for the transport, binding, and release of oxygen. The cytoplasmic ferritin content is regulated by the translation of ferritin H and L mRNAs in response to an intracellular chelatable iron level [41].

Meanwhile the release of free iron by HO-1 increased activity results in upregulation of ferritin and scavenging of the free iron and subsequently plays a central role in cellular antioxidant defense mechanisms [35, 42, 43]. It is known that ferritin plays a key role in maintaining iron homeostasis by capturing and buffering the intracellular labile iron pool [44–46]. Considering that higher ferritin level is associated with the chronic disease, it is not surprising that our cloned pigs show higher mortality. The onset of clinical symptoms of disease and the life span of transgenic mice expressing multiple copies of the human mutant form of the SOD genes are also dependent on the number of transgene copies in their genome [8, 47]. The reduction in transgene copy number resulted in delayed disease onset and an increase in the lifespan of these animals [8]. Therefore, transgene copy number of shTNFRI-Fc and HO-1 might be one of key regulators that mediates liver metabolism.

Taken together, the present study demonstrated that overexpression of shTNFRI-Fc and HO-1 in the pig could increase the transcript levels of iron-ferritin regulated genes and enhance oxidative stress and consequently impair early postneonatal liver metabolism and development. These alterations result in the early onset of severe liver apoptosis and postnatal death. We presented shTNFRI-Fc and HO-1 overexpression piglets as an important model for studying regulation of liver metabolism via oxidative stress but further investigations are required to reveal the molecular pathways of this mechanism.

## Figures and Tables

**Table 1:** Primer sequences used for PCR and real-time RT PCR.

Gene	ID	Primer sequence (5′-3′)		Product size (bp)
GAPDH	NM_001206359	Forward	GTCGGTTGTGGATCTGACCT	124
		Reverse	TTGACGAAGTGGTCGTTGAG	
shTNFRI-Fc^†^	NC_000006.12	Forward	ATA AGCTTATGGGCCTCTCCACCGTGC	633
		Reverse	ATCTCGAGTCATGTGGTGCCTGAGTCCTC	
HO-1^†^	NC_000022.11	Forward	ATGGAGCGTCCGCAACCCGACAG	867
		Reverse	TCACATGGCATAAAGCCCTACAG	
HO-1	NM_002133.2	Forward	TCTCTTGGCTGGCTTCCTTA	109
		Reverse	ATTGCCTGGATGTGCTTTTC	
Ferritin heavy chain	NM_213975.1	Forward	ATGAGCAGGTGAAAGCCATC	110
		Reverse	CAGGGTGTGCTTGTCAAAGA	
Ferritin light chain	NM_001244131.1	Forward	GGAGCGTCTCTTGAAAATGC	125
		Reverse	CCATAGCGTCCTGGGTTTTA	
Transferrin	NM_001244653.1	Forward	GCCACGGAAACCTATTGAGA	126
		Reverse	ACACTGTTGGCACAGTTTGG	
iNOS	NM_001143690.1	Forward	CCACCAGACGAGCTTCTACC	113
		Reverse	TCCTTTGTTACCGCTTCCAC	

^†^Primers for PCR.

**Table 2:** Results of SCNT using *hHAHO-1* overexpressing cells.

Recipient number	Transferred embryo number	Pregnancy	Number of piglets
1	210	No	
2	487	Yes	Abortion
3	300	Yes	Abortion
4	384	Yes	Abortion
5	412	No	
6	416	Yes	7

Total	2209	**4/6 (66.67%)**	
